# Development of a value-based scoring system for the MobQoL-7D: a novel tool for measuring quality-adjusted life years in the context of mobility impairment

**DOI:** 10.1080/09638288.2023.2297929

**Published:** 2024-01-11

**Authors:** Nathan Bray, Rhiannon Tudor Edwards, Paul Schneider

**Affiliations:** a Academy for Health Equity, Prevention and Wellbeing (AHEPW), School of Health Sciences, Bangor University, Bangor, UK; b Centre for Health Economics and Medicines Evaluation (CHEME), School of Health Sciences, Bangor University, Bangor, UK; c School of Health and Related Research (ScHARR), University of Sheffield, Sheffield, UK

**Keywords:** Disability, mobility impairment, quality of life, health-related quality of life, patient reported outcomes, utility, QALY

## Abstract

**Purpose:**

To create a preference-based value set scoring system for the MobQoL-7D outcome measure, and to examine differences in the health state preferences of the general population and individuals with impaired mobility.

**Methods and materials:**

A preference elicitation study was undertaken to ascribe utility weights to all health states (i.e., all unique combination of answers) described by the MobQoL-7D. The elicitation exercise was developed using the Online Elicitation of Personal Utility Functions (OPUF) tool. Two UK sample groups were recruited; firstly a representative general population sample (*N* = 504), secondly a balanced sample of individuals with impaired mobility (*N* = 368). Distinct preference-based value sets were developed for each sample. Differences in dimension ranking, weighting, and overall utility values were assessed.

**Results:**

The general population sample considered most health states, especially the more severe states, to be worse than the mobility impaired sample comparatively. Statistically significant differences between the samples were observed in four of the seven MobQoL-7D dimensions.

**Conclusions:**

This study is the first to provide preference-based value sets for the MobQoL-7D, ready for use in economic evaluations, QALY calculation, and other clinical or research applications. The study demonstrates how the general public and individuals with impaired mobility value health states differently.

## IMPLICATIONS FOR REHABILITATION

- The MobQoL-7D offers a concise and valid tool for rehabilitation professionals to measure and monitor quality of life and quality-adjusted life years (QALYs) in the context of mobility impairment.
- The MobQoL-7D value set calculator allows summary utility scores and QALYs to be calculated using MobQoL-7D outcome data; the first of its kind.
- The general public and individuals with impaired mobility value health states differently, which could impact cost-per QALY calculations and subsequent service commissioning and funding decisions.

## Introduction

The Mobility and Quality of Life 7 Dimension (MobQoL-7D) is a patient-reported measure of health-related quality of life for individuals with impaired mobility. This paper reports the development of a preference-based value set scoring system for the MobQoL-7D, and examines differences in the health state preferences of the general population and individuals with impaired mobility

In 2021/22 24% of the UK population (over 16 million people) reported having some form of disability [1]. Mobility impairment is the second most common cause of disability in the UK, with 43% of disabled people reporting some form of mobility impairment [1], equating to almost 7 million people. The NHS, social care, and third sector provide many different assistive technologies, therapies, and interventions to support people with disabilities and chronic conditions which affect mobility. In each quarter over 700,000 patients are registered with an NHS wheelchair service [2]. An estimated £200million is spent annually by the NHS on manual and powered wheelchair provision alone [3]. More broadly, chronic conditions account for 70% of health and social care expenditure in the UK [4].

Despite the high prevalence of mobility impairment and the associated costs to the public sector and the individual, there is little economic evidence to inform the provision of mobility-enhancing interventions in an evidence-based manner, particularly assistive technology. A systematic review of barriers to using assistive technology for people with long-term conditions found that a lack of evidence causes barriers to influencing government policy, service commissioning, and equipment funding [5].

Public sector resources are limited, particularly following the impact of COVID-19. In order to maximise the value for money of public sector services, evidence is needed to guide decisions about resource allocation. For the economic evaluation of novel and existing health technologies the National Institute for Health and Care Excellence (NICE) recommends the quality-adjusted life year (QALY) approach to outcome measurement [6].

QALYs are calculated using generic (i.e., not condition specific) preference-based measures of health-related quality of life. Preference-based measures are systems of health state classification, where each combination of answers on an outcome measure survey represents a different health state. These measures typically comprise a descriptive system of mutually exclusive health states, and a set of utility values which represent the societal desirability of each health state. Value sets are typically derived from samples of the general public in order to reflect societal preference, however, the importance of incorporating patient preferences is increasingly being recognised, as decisionmakers may also want to assess health benefits from the patients’ perspective. QALYs are calculated by multiplying the amount of time spent in a given health state by the societal desirability of that state [7]. NICE have consistently recommended the EQ-5D outcome measure system for QALY calculation in their technical guidance (6).

A recent systematic review [8] found that preference-based measures often show limited correlation with other clinically relevant outcomes measures associated with physical impairment, reflecting the complex relationship between disability, adaptation, and health-related quality of life. For example, the NICE approved UK value set for the EQ-5D-3L has a disutility of −0.66 for the lowest level of mobility; meaning that an individual who selects the lowest level of mobility on the EQ-5D-3L could achieve a maximum utility value of 0.34 (0 = death; 1 = perfect health), regardless of their outcomes on the other dimensions and any adaptations they may use. Conversely, when suitable adaptations and aids are available many individuals with impaired mobility do not believe that their mobility has a major impact on their health-related quality of life [9, 10].

In the valuation of health state preferences there appears to be a discrepancy between the lived-experience and external perception of disease and disability severity. Individual’s preferences are influenced by the transition from their own health state to the hypothetical health state they are assessing [11–13]. Thus, processes of adaptation are not accounted for when developing values sets from the opinions of the general public [13].

NICE recommend in their reference case that health state preferences should be derived from a representative sample of the general public [6], however guidance in other countries makes a strong argument for using patient preferences instead [14]. There continues to be debate about whose preferences should guide decision-making though [15]. Evidence shows that preferences differ between the general public and patient groups and that this has an impact on subsequent utilities and cost-effectiveness estimates [14].

Condition-specific preference-based measures and patient-elicited value sets have greater sensitivity in specific patient-groups, at the expense of genericity [15, 16]. Such measures are typically developed to cover the key dimensions of health and quality of life associated with a particular condition or disease and are thus more sensitive and responsive to what matters to those individuals who are affected by a particular health issue. Over 50 condition-specific preference-based measures have been developed [17], including the MobQoL-7D for mobility-related quality of life [18, 19].

The descriptive system of the MobQoL-7D comprises seven dimensions of mobility-related quality of life which are broadly relevant to all forms of mobility impairment [19]. The development of the MobQoL-7D consisted of two previous studies;

1. An exploratory descriptive study using a qualitative framework analysis approach to generate a “de novo” (i.e., developing from new) outcome measure for mobility-related quality of life [18]; *n* = 37 individuals with impaired mobility, publication year 2020.
2. A cross-sectional methodological study using psychometric analysis, factor analysis and Rasch analysis to derive a novel health state classification system from the initial MobQoL tool [19]; *n* = 342 individuals with impaired mobility, publication year 2022.

Principles of preference-based outcome measurement informed both item development and item selection of the MobQoL-7D.

The MobQoL-7D comprises two underlying factors within the item structure: (1) physical and role functioning related to mobility, and (2) mental wellbeing related to mobility. Rasch analysis confirmed that these two factors represent two unidimensional sub-scales. The measure contains seven dimensions, each representing a distinct conceptual dimension of mobility-related quality of life: Accessibility (AC), Contribution (CO), Pain/Discomfort (PD), Independence (IN), Self-Esteem (SE), Mood/Emotions (ME), and Anxiety (AX).

This paper presents a quantitative survey-based preference elicitation exercise to elicit utility weights for all health states described by the MobQoL-7D, for the purpose of creating a preference-based value set scoring system for this outcome measure.

### Aims and objectives

The aim of this study was to create a preference-based value set scoring system for the MobQoL-7D, and to examine differences in the health state preferences of the general population and individuals with impaired mobility.

## Materials and methods

The study was conducted in September 2022 and was ethically approved by Bangor University’s School of Medical and Health Sciences ethics committee (reference no. 2022-17202). The project was funded by the Welsh Government through Health and Care Research Wales as part of their Health Fellowship scheme [HF-16-1159] and supported by The Wellcome Trust [108903/B/15/Z].

Participants were identified through the online research participant platform Prolific (https://www.prolific.co/), who have been shown to be an effective source of recruitment for research [20]. Prolific provide nationally representative samples and have a specific sub-sample of UK individuals who identify as having a “physical disability/reduced mobility”.

Two samples were recruited: a general population sample (GP) and a mobility impaired sample (MI). The GP sample was stratified by age, gender, and ethnicity in order to be representative of the UK population in these key demographics. The MI sample was balanced by gender; further stratification using age and ethnicity was not possible due to the limited sampling pool available for individuals with impaired mobility. In order to identify participants for the MI sample two techniques were used: Firstly, Prolific’s sample stratification criteria were used to identify individuals with long-term health conditions or disabilities which resulted in reduced mobility; secondly, participants were asked if they had been diagnosed with an injury, disability or health condition which affected their mobility, and which resulted in problems with their mobility lasting for more than 6 months; any individual answering yes to this question was included in the MI sample.

Potential participants accessed information about the study through Prolific’s proprietary online recruitment system and were then directed to an online survey developed for the project. The online survey was the primary method of data collection. Before completing the survey all participants were presented with an information sheet about the project and completed a consent form. In line with good practice all participants were offered a small financial incentive (£2.50) for taking part.

**Figure A1.**
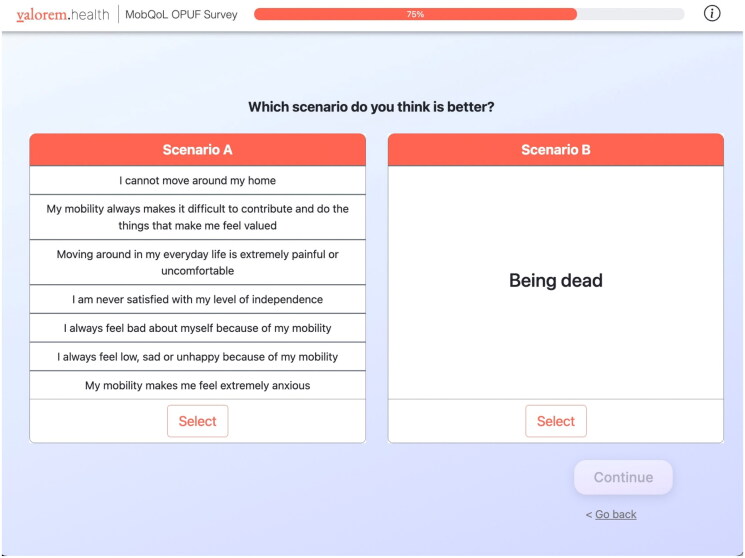
Participants were asked to consider a pairwise comparison between the worst MobQoL-7D health state “444444” (scenario A; a state in which all seven dimensions are at the worst level) and ‘being dead’ (scenario B).

**Figure A2.**
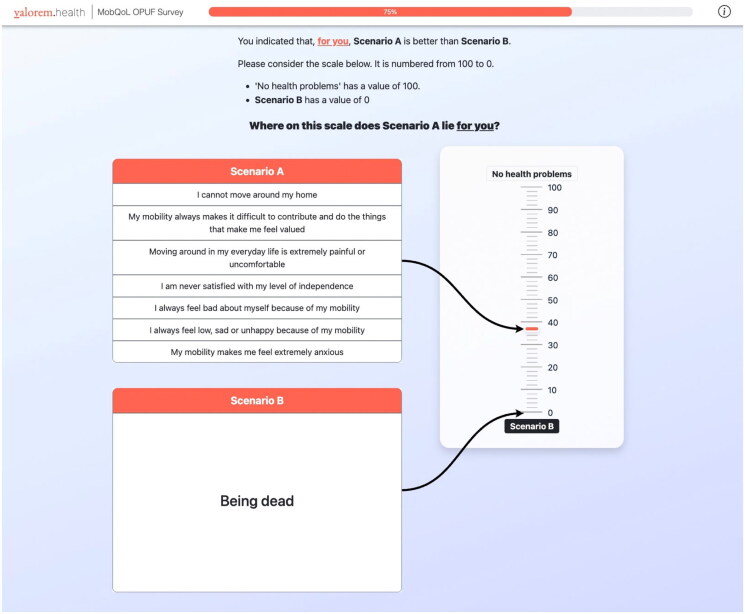
If participants preferred the scenario A (the worst health state), over “being dead” in the S1task, participants were asked to locate the position of scenario A on a VAS between “no mobility problems” (=100) and “being dead” (=0).

**Figure A3.**
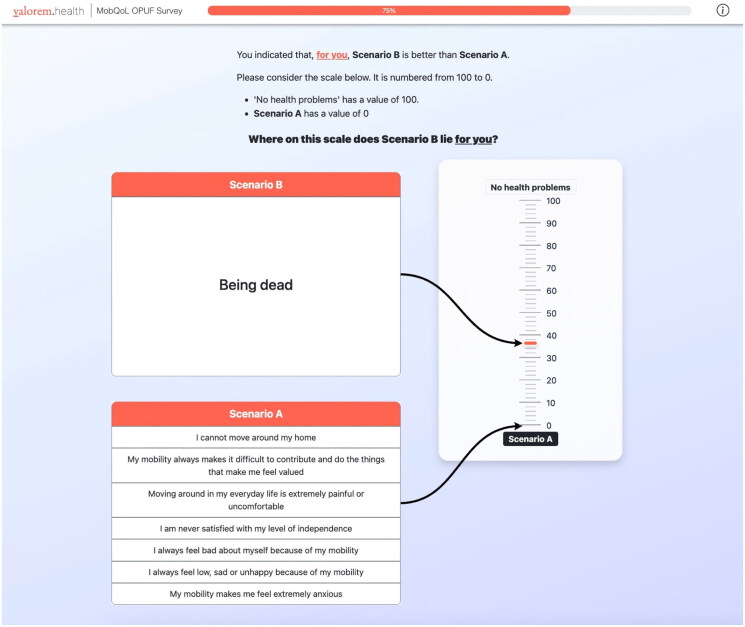
If participants indicated that they preferred “being dead” over scenario A in the S1 task, they were asked to locate the position of “being dead” on a VAS between “no mobility problems” (=100) and scenario A (=0).

The MobQoL-7D preference elicitation survey was developed using the Online Elicitation of Personal Utility Functions (OPUF) tool [21]. The OPUF-based survey consisted of five tasks which participants undertook sequentially:

1. *Completion of MobQoL-7D*: Participants were asked to complete the MobQoL-7D to report their own state of mobility-related quality of life. Participants were also asked to provide basic demographic information.
2. *Dimension selection*: Participants were shown the worst level of each MobQoL-7D dimension (e.g., “I cannot move around my home” for Accessibility, “I am never satisfied with my level of independence” for Independence, etc.), and asked to indicate which of these problems would have or has the most negative impact on their lives. The dimension selected by the participant as having the most impact was then used to tailor the comparator dimension in task 3 (see below for further information).
3. *Dimension swing weighting*: Participants were presented with sliders for each MobQoL-7D dimension, describing an improvement from the worst to the best level on the respective dimension (e.g., “I am never satisfied with my level of independence” to “I am always satisfied with my level of independence”). The first slider (the most impactful dimension from task 2) was set to 100, and the participants were asked to use this as a yardstick to evaluate the importance of the remaining dimensions, which were shown and assessed individually, in a random order.
4. *Level rating*: The level ratings of each dimension were elicited by asking participants to position the intermediate levels (level 2 and level 3) on a visual analogue scale (VAS), anchored between the worst (i.e., 0%) and best (i.e., 100%) level of each dimension. For the “Independence” dimension, for example, the VAS was anchored at “I am never satisfied with my level of independence” (=0%) and “I am always satisfied with my level of independence” (=100%), and participants were then asked to indicate where on this scale they think the levels “I am often satisfied with my level of independence” (level 2) and “I am sometimes satisfied with my level of independence” (level 3) lie.
5. *Anchoring:* Participants were asked to consider a pairwise comparison between the worst MobQoL-7D health state “444444” (scenario A; a state in which all seven dimensions are at the worst level) and “being dead” (scenario B) (see Figure A1 in the appendix). If participants preferred scenario A (the worst health state), over “being dead”, participants were asked to locate the position of scenario A on a VAS between “no mobility problems” (=100) and “being dead” (=0) (see Figure A2 in the appendix). If they indicated that they preferred “being dead” over scenario A, they were asked to locate the position of “being dead” on a VAS between “no mobility problems” (=100) and scenario A (=0) (see Figure A3 in the appendix). The response to this task was used to rescale and anchor the personal utility function so that it could be mapped on to a 0 to 1 QALY-style scale.

Personal utility functions were constructed within the OPUF system for all participants using the data they provided in the survey. The levels were rescaled between 0 (best level) and 1 (worst level). The seven dimension weights were normalised to sum 1. The outer product of the dimension weights and the level ratings were taken to generate a set of 21 (un-anchored) model coefficients (+7 zero coefficients). The response from the Anchoring task (task 5) was used to rescale the model coefficients and map them on to the QALY-style scale. Finally, the model coefficients were used to generate utility values for all possible MobQoL-7D health states – this vector of utility values represents the personal utility functions. Health states are referred to by their 7-digit dimension-level indices; these are simple representations of an individual’s answers on the MobQoL-7D and thus indicative of their health state. For instance, if an individual were to state that they had no problems (i.e., choose response level 1) on all dimensions, their health state would be “1111111”, if they were to state that they had extreme problems (i.e., chose response level 4) on all dimensions, their health state would be “4444444”, and so on. Each potential combination of answers is represented by a unique 7-digit health state index value.

For a full explanation of how the OPUF system works see the original paper by Schneider et al. [21].

Separate MobQoL-7D value sets were generated for the two samples, i.e., one for the general UK population, and one for individuals in the UK with mobility impairments. In order to examine the differences between the two samples a number of factors were taken into consideration:

- Level ratings: Comparison of sample preferences for different levels of each dimension
- Dimension selection: Comparison of the distribution of weights assigned to each dimension and subsequent dimension rankings
- Model coefficients: Comparison of differences in the mean model coefficient estimates, and identification of any statistically significant differences
- Anchoring: Comparison of the total number (%) of health states valued as worse than death
- Value sets: Comparison of preference weights for a range of health states, including those at the extreme ends of the utility scale, and differences in the total value ranges of the two value sets

Finally, plots were produced to illustrate the distribution of preference weights for all health states for both samples.

## Results

### Demographics

In total, 504 participants were included in the GP sample and 368 participants in the MI sample. The GP sample was broadly representative of the UK population in terms of gender, ethnicity and age according to recent demographic data [22]. Prolific stratify age using five 9-year brackets: 18–27, 28–37, 38–47, 48–57, and 58+, however age brackets are presented in Table 1 to comply with UK census reporting. Comparable UK demographics specifically for individuals with impaired mobility were not available. Gender distribution for both samples was all but equal, and both samples were predominantly white (GP = 85.9%; MI = 95.4%). In the MI sample, 54.1% of participants were aged >50 years, compared to 43.5% in the GP sample. The proportion of participants in paid employment was higher in the GP sample (57.1%) compared to the MI sample (49.7%), possibly due to the differences in age groups. See Table 1 for full demographic details.

**Table 1.** Demographics by sample.

		GP *N* = 504		MI *N* = 368	
		*N*	%	*N*	%
Gender	Male	244	48.41	179	48.64
	Female	256	50.79	180	48.91
	Other	1	0.20	8	2.17
	Not stated	3	0.60	1	0.27
Age	18–29	99	19.64	34	9.24
	30–39	94	18.65	69	18.75
	40–49	92	18.25	66	17.93
	50–59	103	20.44	93	25.27
	60–69	95	18.85	88	23.91
	70+	15	2.98	16	4.35
	Not stated	6	1.19	2	0.54
Ethnicity	White	433	85.91	351	95.38
	Black	16	3.17	3	0.82
	Asian	34	6.75	2	0.54
	Mixed	11	2.18	8	2.17
	Other	10	1.98	4	1.09
Employment	Full-time	209	41.47	114	30.98
	Not in paid work	89	17.66	129	35.05
	Part-Time	79	15.67	69	18.75
	Not stated	90	17.86	30	8.15
	Unemployed	16	3.17	14	3.80
	Other	19	3.77	11	2.99
	Due to start a new job	2	0.40	1	0.27

### Mobility impairment

Upon completion of the MobQoL-7D, the MI sample exhibited far greater severity of mobility impairment, with 28% (*N* = 72) of the sample indicating severe problems on at least one MobQoL-7D dimension, compared to 2% (*N* = 11) of the GP sample. None of the MI sample reported having “no problems” on any of the MobQoL-7D dimensions, compared to 47% (*N* = 238) of the GP sample. Mean (SD) severity scores were 9.3 (3.2) and 16.7 (4) for the GP and MI samples respectively. These results were expected and demonstrate appropriate sampling methods.

### Level ratings

The level ratings reflect sample preferences for different levels of each dimension, which in turn illustrate how much better one level is perceived to be to another, for instance “slight pain” versus “extreme pain”. The levels for each dimension reflect severity, where 1 = no problems, 2 = some problems, 3 = moderate/severe problems and 4 = extreme problems. The scale is defined by the best and worst possible level of severity, hence values have been normalised between 100 (best) and 0 (worst), with the ratings of “no problems” (i.e., level 1 for each dimension) and “extreme problems” (i.e., level 4 for each dimension) fixed at 100 and 0 respectively. See Table 2 for all level rating results. For both samples, level 2 for the Independence dimension had the lowest mean (SD) severity rating, at 61.9 (26.8) for the MI sample and 72.9 (18.7) for the GP sample. The dimension which had the highest mean (SD) severity rating at the “moderate/severe problems” level (i.e., level 3) was Pain/Discomfort (32 [27]) for the MI sample and Anxiety (29 [24.2]) for the GP sample.

**Table 2.** Level rating by MobQoL-7D dimension.

MobQoL-7D Dimension^a^	GP lvl2 mean (SD)	MI lvl2 mean (SD)	GP lvl3 mean (SD)	MI lvl3 mean (SD)
AC	57.1 (24.9)	55.2 (24.3)	29.6 (26.5)	32.6 (26.8)
CO	55.2 (22.3)	53.5 (21.4)	32.9 (22.9)	34.8 (22.9)
PD	57.5 (24.8)	56.7 (24.1)	30.9 (28.2)	32 (27)
IN	72.9 (18.7)	61.9 (26.8)	43.9 (20)	41.6 (19)
SE	53 (24.5)	53.6 (22)	32.9 (24.2)	35.2 (24.9)
ME	52.9 (22.8)	54.1 (21.8)	32 (23.2)	34.2 (24.6)
AX	55.5 (26)	54.4 (24.8)	29 (24.2)	32.2 (25.3)

AC: Accessibility; CO: Contribution; PD: Pain/Discomfort; IN: Independence; SE: Self-Esteem; ME: Mood/Emotions; AX: Anxiety.

^a^ The ratings of “no problems” (i.e., level 1 for each dimension) and “extreme problems” (i.e., level 4 for each dimension) are fixed at 100 and 0 respectively.

### Dimension weighting/ranking

The dimension weighting tasks yield a distribution of weights assigned to each of the seven MobQoL-7D dimensions, which subsequently reveal the relative importance of each dimension for individual health state preference. See Table 3 for all dimension weighting and ranking results. The dimension with the highest mean (SD) weight was Pain/Discomfort for both the GP and MI samples at 88.3 (20.3) and 86.6 (22.7) respectively, indicating that this dimension was of most importance. Likewise the dimension with the second highest weighting for both samples was Accessibility (84.7 [23.1] and 79 [26.5] respectively). All other dimensions varied in their ranking between the two samples. For the MI sample, the least important dimension was Anxiety (61.3 [31.6]), while for the GP sample the least important dimension was Self-Esteem (62.7 [31.2]).

**Table 3.** MobQoL-7D dimension weighting and ranking.

MobQoL-7D dimension	GP weighting mean (SD)	GP Rank	MI weighting mean (SD)	MI Rank
AC	84.7 (23.1)	2	79 (26.5)	2
AX	64.3 (30.9)	6	61.3 (31.6)	7
CO	71.9 (27.2)	5	76.6 (26.7)	3
IN	73.9 (26.8)	3	72.4 (27.7)	4
ME	72 (28.2)	4	70.4 (28.7)	5
PD	88.3 (20.3)	1	86.6 (22.7)	1
SE	62.7 (31.2)	7	62.2 (29.8)	6

AC: Accessibility; CO: Contribution; PD: Pain/Discomfort; IN: Independence; SE: Self-Esteem; ME: Mood/Emotions; AX: Anxiety.

### Model coefficients

Table 4 shows the differences (GP-MI) in the mean coefficients for each dimension level, with bootstrapped 95% confidence intervals (based on 10,000 iterations). Coefficients are significantly different (at the 5% level) if the 95% confidence interval does not cross 0. Statistically significant differences were observed at level 3 and 4 for the Accessibility dimension (0.021 [0.009; 0.034] and 0.020 [0.003; 0.037] respectively), level 3 for the Mood/Emotions dimension (0.014 [0.003; 0.025]) and level 4 for the Pain/Discomfort (0.021 [0.002; 0.040]) and Independence (0.016 [0.002; 0.030]) dimensions.

**Table 4.** Mean MobQoL-7D model coefficient differences (95% confidence intervals) between the GP and MI samples.

MobQoL-7D dimension level	AC	CO	PD	IN	SE	ME	AX
lvl2	0.007 (−0.004; 0.017)	−0.001 (−0.010; 0.007)	0.007 (−0.005; 0.019)	−0.005 (−0.012; 0.001)	0.001 (−0.008; 0.008)	0.005 (−0.003; 0.014)	0.003 (−0.005; 0.010)
lvl3	0.021* (0.009; 0.034)	−0.000 (−0.013; 0.012)	0.012 (−0.003; 0.027)	0.008 (−0.001; 0.017)	0.005 (−0.005; 0.015)	0.014* (0.003; 0.025)	0.009 (−0.001; 0.019)
lvl4	0.020* (0.003; 0.037)	−0.002 (−0.019; 0.014)	0.021* (0.002; 0.040)	0.016* (0.002; 0.030)	0.001 (−0.013; 0.014)	0.009 (−0.007; 0.025)	0.008 (−0.006; 0.020)

*Significant difference at 5% level.

Coefficient difference calculated as GP mean minus MI mean.

AC: Accessibility; CO: Contribution; PD: Pain/Discomfort; IN: Independence; SE: Self-Esteem; ME: Mood/Emotions; AX: Anxiety.

### Anchoring

In the GP group, 331 (66%) participants indicated they would prefer the worst MobQoL-7D health state (i.e., 4444444; extreme problems across all dimensions) over being dead. The MI group exhibited similar findings, although had a stronger preference for the worst state over death, with 181 (72%) participants indicating that they would prefer the worst state over being dead. On average both groups considered all health states better than being dead.

After capping the utility scale at −1, the mean (SD) anchor point (i.e., the utility of the worst health state) was 0.171 (0.472) in the GP group, and 0.242 (0.47) in the MI group. In the GP group, only 29 (6%) participants had anchor points below −1. Similarly, in the MI group, there were only 14 (6%) participants with anchor points below −1.

### Value sets

**Figure 1.**
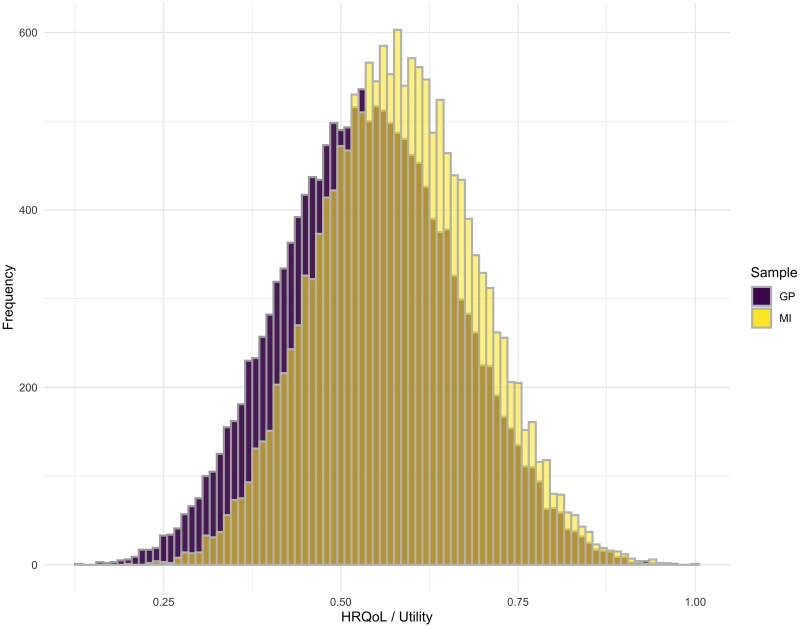
Frequency of utility values across all MobQoL-7D health states, by sample. On the X axis the utility space is plotted from 0 to 1, where 1 represents the best possible health state and 0 represents the worst possible health state. On the Y axis the frequency of health states is plotted. Comparing the MI sample depicted in yellow, and the GP sample depicted in purple, we see that the MI sample rated more health states higher than the GP sample, particularly between utility values of 0.50 and 0.75, and were less likely to rate health states as having utility values below 0.50.

**Figure 2.**
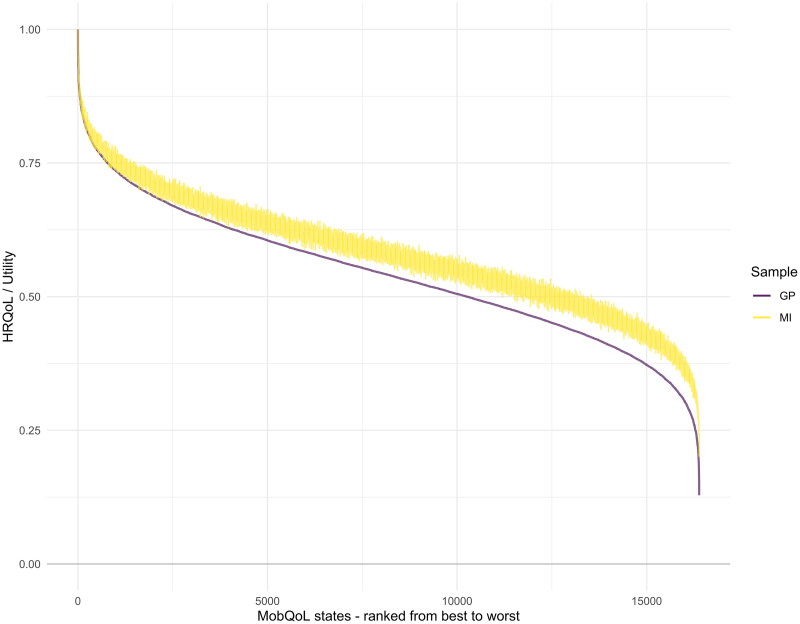
Distribution of MobQoL-7D utility values from best (i.e., 1111111) to worst (i.e., 4444444), by sample. On the X axis all 16,384 MobQoL-7D health states are plotted form the worst (i.e., 4444444) to the best (i.e., 1111111). On the Y axis the utility values for each health state are plotted, from 1 (highest possible utility value) to 0 (lowest possible utility value). Comparing the MI sample depicted in yellow, and the GP sample depicted in purple, we see that the MI sample rated most health states higher than the GP sample. The differences between the two samples become more apparent as the health states worsen. The GP data line appears smooth because health states are ordered along the x-axis according to the preference ordering of the GP sample; the MI data line shows variance (compared to GP sample) due to the different relative ranking of health states.

See Tables 5 and 6 for the final value sets for each sample. A utility value calculator has been developed using these value sets to calculate summary scores and QALY outcomes from MobQoL-7D data; see supplemental file 1 to access the calculator. The MI value set consistently values MobQoL-7D states higher than the GP value set, this is illustrated in Figures 1 and 2 which show the frequency and distribution of utility values across all health states, on a scale from 0 (death) to 1 (perfect health). The lowest possible utility value on the MobQoL-7D is 0.129 using the GP value set and 0.202 using the MI value set.

**Table 5.** Final MobQoL-7D value set, derived from a representative general population sample – presenting mean (95% confidence intervals) utility value per dimension and level.

MobQoL-7D dimension level	AC	CO	PD	IN	SE	ME	AX
lvl2	0.063 (0.057; 0.070)	0.054 (0.049; 0.059)	0.068 (0.061; 0.076)	0.033 (0.030; 0.037)	0.046 (0.042; 0.050)	0.055 (0.050; 0.060)	0.044 (0.040; 0.049)
lvl3	0.106 (0.097; 0.116)	0.082 (0.075; 0.089)	0.111 (0.102; 0.122)	0.072 (0.066; 0.078)	0.068 (0.062; 0.074)	0.084 (0.076; 0.092)	0.073 (0.067; 0.079)
lvl4	0.148 (0.138; 0.159)	0.121 (0.112; 0.130)	0.160 (0.148; 0.173)	0.124 (0.115; 0.134)	0.098 (0.091; 0.105)	0.119 (0.110; 0.129)	0.101 (0.094; 0.109)

AC: Accessibility; CO: Contribution; PD: Pain/Discomfort; IN: Independence; SE: Self-Esteem; ME: Mood/Emotions; AX: Anxiety.

**Table 6.** Final MobQoL-7D value set, derived from a balanced sample of individuals with impaired mobility – presenting mean (95% confidence intervals) utility value per dimension and level.

MobQoL-7D dimension level	AC	CO	PD	IN	SE	ME	AX
lvl2	0.056 (0.049; 0.065)	0.055 (0.049; 0.062)	0.061 (0.052; 0.070)	0.038 (0.033; 0.044)	0.045 (0.039; 0.052)	0.050 (0.043; 0.057)	0.042 (0.036; 0.048)
lvl3	0.085 (0.076; 0.094)	0.082 (0.072; 0.093)	0.099 (0.088; 0.111)	0.064 (0.057; 0.071)	0.063 (0.056; 0.072)	0.070 (0.063; 0.079)	0.064 (0.056; 0.072)
lvl4	0.128 (0.115; 0.141)	0.123 (0.109; 0.138)	0.139 (0.126; 0.154)	0.108 (0.098; 0.119)	0.098 (0.087; 0.109)	0.110 (0.098; 0.122)	0.093 (0.083; 0.105)

AC: Accessibility; CO: Contribution; PD: Pain/Discomfort; IN: Independence; SE: Self-Esteem; ME: Mood/Emotions; AX: Anxiety.

Utility values for the top 25 and bottom 25 ranked health states are presented in Table 7. The differences in utility values in the more extreme states reveal meaningful discrepancies between the two samples. In the bottom 25 health states, which have the lowest utility scores, the mean (SD) difference in utility between the MI and GP samples was −0.073 (0.005), with all health states exhibiting lower utility values in the GP sample. Looking across all of the 16,384 possible MobQoL-7D health states, the mean (SD) difference in utility between the samples was −0.039 (0.016), which would reflect a minimally important difference by the standard of other utility measures [23]. The discrepancy between the samples increases as the health states worsen, with a 25th percentile of −0.028, a 50th percentile −0.039, and a 75th percentile of −0.050. Overall this indicates that comparatively the GP sample considered most health states, especially the more severe states, to be worse than the MI sample did.

**Table 7.** Utility values for top 25 and bottom 25 ranked MobQoL-7D health states.

MobQoL-7D health state	GP utility	MI utility	Utility difference	GP rank	MI rank	Rank difference
1111111	1.000	1.000	0.000	1	1	0
1112111	0.967	0.962	0.005	2	2	0
1111112	0.956	0.958	−0.003	3	3	0
1111211	0.954	0.955	−0.001	4	4	0
1211111	0.946	0.945	0.001	5	6	−1
1111121	0.945	0.950	−0.005	6	5	1
2111111	0.937	0.944	−0.007	7	7	0
1121111	0.932	0.939	−0.007	8	8	0
1111311	0.932	0.937	−0.005	9	9	0
1113111	0.928	0.936	−0.008	10	11	−1
1111113	0.927	0.936	−0.009	11	10	1
1112112	0.923	0.920	0.003	12	13	−1
1112211	0.921	0.916	0.005	13	15	−2
1311111	0.918	0.918	0.000	14	14	0
1111131	0.916	0.930	−0.014	15	12	3
1212111	0.913	0.906	0.007	16	21	−5
1112121	0.912	0.912	0.000	17	18	−1
1111212	0.910	0.913	−0.003	18	17	1
2112111	0.904	0.905	−0.002	19	22	−3
1211112	0.902	0.903	−0.001	20	24	−4
1111411	0.902	0.902	−0.001	21	25	−4
1111122	0.901	0.909	−0.008	22	19	3
1211211	0.900	0.899	0.001	23	29	−6
1111221	0.899	0.905	−0.006	24	23	1
1111114	0.899	0.907	−0.008	25	20	5
4444243	0.209	0.283	−0.074	16,360	16,354	6
4443443	0.209	0.275	−0.067	16,361	16,364	−3
4434344	0.207	0.275	−0.068	16,362	16,363	−1
4434443	0.205	0.271	−0.066	16,363	16,370	−7
3444434	0.205	0.283	−0.078	16,364	16,355	9
4344434	0.202	0.281	−0.078	16,365	16,357	8
3444344	0.200	0.277	−0.078	16,366	16,361	5
3444443	0.198	0.273	−0.075	16,367	16,366	1
4344344	0.197	0.276	−0.078	16,368	16,362	6
4244444	0.195	0.268	−0.073	16,369	16,372	−3
4344443	0.195	0.271	−0.076	16,370	16,368	2
4444334	0.193	0.274	−0.081	16,371	16,365	6
4444424	0.193	0.261	−0.068	16,372	16,374	−2
4444433	0.191	0.270	−0.078	16,373	16,371	2
4444343	0.186	0.265	−0.078	16,374	16,373	1
4444442	0.185	0.253	−0.067	16,375	16,376	−1
4444244	0.181	0.253	−0.072	16,376	16,375	1
4443444	0.181	0.245	−0.065	16,377	16,377	0
4434444	0.178	0.241	−0.064	16,378	16,380	−2
3444444	0.170	0.243	−0.073	16,379	16,378	1
4344444	0.167	0.241	−0.074	16,380	16,379	1
4444434	0.164	0.240	−0.077	16,381	16,381	0
4444344	0.158	0.235	−0.077	16,382	16,382	0
4444443	0.156	0.230	−0.074	16,383	16,383	0
4444444	0.128	0.201	−0.072	16,384	16,384	0

Health state indices, obtained from participants before the valuation tasks, and estimated health state utility values from both the GP and the MI value set, were used to calculate MobQoL-7D population norms by age and gender. The results are presented in Tables 8 and 9. Health state information from participants who did not provide gender and age information were excluded from the analysis.

**Table 8.** MobQoL-7D population norms by age and gender, for the MI sample based on the GP^a^ and MI^b^ value sets.

Gender	Age	Mean^a^	Mean^b^	95% CI	*N*
Female	18 − 29	0.512	0.551	(0.416; 0.608)	8
	30 − 39	0.503	0.545	(0.464; 0.543)	24
	40 − 49	0.558	0.593	(0.500; 0.616)	23
	50 − 59	0.538	0.577	(0.491; 0.586)	27
	60 − 69	0.57	0.603	(0.519; 0.62)	30
	70+	0.581	0.612	(0.422; 0.739)	6
Male	18 − 29	0.57	0.607	(0.499; 0.642)	11
	30 − 39	0.533	0.569	(0.472; 0.595)	23
	40 − 49	0.61	0.64	(0.545; 0.675)	24
	50 − 59	0.522	0.562	(0.457; 0.587)	31
	60 − 69	0.587	0.618	(0.537; 0.638)	32
	70+	0.719	0.733	(0.589; 0.848)	4

^a^ Calculated using GP value set.

^b^ Calculated using MI value set.

**Table 9.** MobQoL-7D population norms by age and gender for the GP sample, based on the GP value set.

Gender	Age	Mean^a^	95% CI	*N*
Female	18 − 29	0.919	(0.886; 0.952)	50
	30 − 39	0.897	(0.855; 0.938)	46
	40 − 49	0.856	(0.815; 0.897)	45
	50 − 59	0.845	(0.793; 0.896)	55
	60 − 69	0.882	(0.842; 0.922)	49
	70+	0.834	(0.710; 0.959)	7
Male	18 − 29	0.951	(0.924; 0.978)	48
	30 − 39	0.887	(0.846; 0.929)	47
	40 − 49	0.914	(0.866; 0.963)	46
	50 − 59	0.848	(0.797; 0.899)	48
	60 − 69	0.882	(0.844; 0.919)	46
	70+	0.913	(0.837; 0.989)	8

^a^ Calculated using GP value set.

## Discussion

This paper presents two preference-based value sets for the MobQoL-7D outcome measure; one from a representative sample of the UK general public, and one from a balanced sample of UK individuals with impaired mobility. The dissimilarities between the value sets reveal important differences in the health state preferences of the general public compared to individuals with impaired mobility.

In this study the GP sample considered most health states, especially the more severe states, to be worse than the MI sample comparatively (see Figures 1 and 2). The average difference in utility between the samples was −0.039 (SD = 0.016), which likely represents a minimally important difference (i.e., a difference which would likely be considered meaningful to a patient) [23]. This could be due to a number of factors, including processes of habituation and adaptation experienced by the MI sample, or the way in which individuals tend to focus on the transition from their own health state to a hypothetical health state [11–13].

A key consideration is whether to use the MI value set or the GP value set when calculating outcomes from the MobQoL-7D. Due to differences in the way the two samples ranked and weighted the MobQoL-7D dimensions and levels, it is unsurprising that the final value sets vary. As noted above, the differences in utility values between the value sets are likely to be meaningful, highlighting the importance of considering perspective when using health state preferences.

Preference-based utility measures are a cornerstone of health economics, as they allow societal preferences to inform the evaluation of the cost-effectiveness of new and existing health technologies [7]. This is particularly important when considering cost-effectiveness in the context of tax-funded national health services, where accountability for the spending of public money is highly relevant.

There is significant debate about who’s preferences matter in health state valuation, and there are compelling arguments for both patient and societal preferences [15]. On one hand, NICE advise that a value set should represent societal preferences more broadly to enable comparability across disparate interventions and services [6], hence the ubiquity of this approach particularly in the UK. On the other hand, patients are the key stakeholders in their own health and health care and thus have the most relevant real-world experience to value related health states [24].

It is our belief that both approaches have merits and that the intended use of the MobQoL-7D outcome measure should determine the approach to health state valuation. Where patient experience is of key importance the MI value set should be used, for example when measuring patient-reported outcomes. Where comparability, cost-effectiveness, and broader societal preferences are paramount, the GP value set could be more appropriate as a primary source, with the MI value set considered as a secondary source of additional value information.

In the applied use of the MobQoL-7D, the differences between the outcomes of these two value sets are likely to be highly informative. Thus using one value set in a base case analysis and the other in a subsequent sensitivity analysis could demonstrate interesting and important variance in the outcomes.

It is important to consider the application and usefulness of QALY data derived from the MobQoL-7D. The OPUF approach produces a scale anchored at 0 (dead) and 1 (full health), and thus OPUF value sets can be used to calculate QALYs. The MobQoL-7D focusses specifically on mobility-related quality of life and is therefore conceptually different to generic health-related quality of life measures like the EQ-5D-5L. The MobQoL-7D should be used to measure the extent to which an individual’s mobility-related quality of life affects their utility. The comparability of a QALY derived from a generic measure and a QALY derived from a function or condition-specific measure is debatable [6]. Bodies such as NICE still have a specific methodological preference for generic measures in this context, despite a potential lack of sensitivity in certain patient groups [13, 24]. It is therefore important to consider the trade-off between genericity and sensitivity when using preference-based outcome measures, and whether these two different approaches should be considered complementary or mutually exclusive.

The OPUF approach to preference elicitation was chosen due the ease with which it can be applied and the relative statistical power. Compared to traditional methods of preference elicitation, such as Time Trade-Off (TTO) and Standard Gamble (SG), the OPUF approach requires far fewer participants to derive a group tariff or social value set with similar precision around mean estimates, making it specifically useful for estimating value sets for patient groups and other demographic sub-groups [21]. This study also demonstrates that an online tool like the OPUF can be used effectively to elicit personal and group-level preferences for different health states and that this approach can be completed entirely online without assistance. This makes it substantially easier and more affordable than approaches like TTO, which typically require guided interviews to collect data due to the complexity of the tasks. Furthermore, previous research has demonstrated the possible equivalence of online and in-person preference elicitation methods [25]. This paper illustrates that the OPUF approach can be applied to a novel condition-specific patient reported outcome measure, to derive individual and group-level preference-weights.

There are limitations to this study which require consideration: firstly, due to the self-selective nature of recruitment for online research, the samples may lack the representativeness of more marginalised groups. Both samples lack representation for individuals aged 70 years or older, despite generally being representative of the UK population in terms of the proportion of individuals aged under or over 50 years. Furthermore, it is likely that the recruitment strategy did not yield a sample that is truly representative of individuals with impaired mobility in the UK. Secondly the OPUF approach and online system is still relatively new and therefore may be less refined than traditional preference elicitations methods. Thirdly the OPUF preference elicitation exercise is reasonably complex and entirely undertaken online; thus we did not have the opportunity to check that participants fully understood the task prior to data collection. In order to counter this potential issue, all data were checked for errors and inconsistencies prior to inclusion in analyses; 11 participants’ data were accordingly removed from analyses due to issues such as rating the best possible health state worse than the worst possible health state.

Future research should utilise the MobQoL-7D to examine differences between relevant patient groups, for instance individuals with congenital mobility impairments compared to individuals with acquired mobility impairments. Further work is needed to understand the extent of comparability and agreement between the MobQoL-7D and other preference-based measures and condition specific measures.

To conclude, we have developed the first preference-based value sets for the MobQoL-7D outcome measure. The value sets will be made available for general use alongside the MobQoL-7D outcome measure, available here: https://cheme.bangor.ac.uk/mobqol. This study demonstrates how the general public and individuals with impaired mobility value health states differently, and the impact this may have on utility-based QALY outcomes and subsequent cost-per QALY calculations; this could in turn have significant impacts on service commissioning and funding decisions for people living with mobility impairments. It is therefore important to consider perspective when using preferences to determine the desirability of different health states. The OPUF approach is a feasible and expedient method of developing health states preferences through online data collection.

## Supplementary Material

Supplemental File1 MobQoL-7D Calculator v1.0 Oct22.xlsx

## Data Availability

The underlying anonymised data is freely available from: https://doi.org/10.5281/zenodo.7344501 Data are available under the terms of the Creative Commons Zero “No rights reserved” data waiver (CC0 1.0 Public domain dedication).
